# Applying Outdoor Environment to Develop Health, Comfort, and Energy Saving in the Office in Hot-Humid Climate

**DOI:** 10.1155/2013/367283

**Published:** 2013-11-07

**Authors:** Rong Chen, Wen-Pei Sung, Hung-Chang Chang, Yi-Rou Chi

**Affiliations:** ^1^College of Civil and Architectural Engineering, Wuyi University, Wuyi, Fujian 354300, China; ^2^Department of Landscape Architecture, National Chin-Yi University of Technology, Taichung 411, Taiwan; ^3^Department of Civil Engineering, Chung Hua University, Hsinchu 300, Taiwan

## Abstract

A human life demand set to emerge in the future is the achievement of sustainability by maintaining a comfortable indoor environment without excessive reliance on energy-consuming air conditioners. The major research processes in this study are: (1) measuring indoor air quality and thermal comfort to evaluate the comfort of an indoor environment; (2) implementing questionnaire survey analysis to explore people's environmental self-perceptions and conducting a meta-analysis of the measurement results for air quality and physical aspects; and (3) constructing an indoor monitoring and management system. The experimental and analysis results of this research reveal that most of the office occupants preferred a cooler environment with a lower temperature. Additionally, because the summers in Taiwan are humid and hot, the occupants of an indoor space tend to feel uncomfortable because of the high humidity and poor indoor air quality. Therefore, Variable Air Volume (VAV), two air intakes, and exhaust plant are installed to improve indoor environment. After improvement, a lower temperature (approximately 21.2–23.9°C) indirectly reduces humidity, thereby making the occupants comfortable. Increasing air velocity to 0.1 ~ 0.15 m/s, the carbon dioxide concentrations decrease below the requirement of the WHO. Ninety-five percent of the workers corresponded to the standard comfort zone after this improvement.

## 1. Introduction

With the continuous development of technology, centralization of populations has contributed to the trend of urbanization. The earth's surface has been affected by artificial developments, and people's living spaces have expanded. This has increased people's metabolic heat and energy consumption, which has affected thermal equilibrium in urban areas. Additional problems include energy poverty, the destruction of natural environments, and global climate anomalies. Environmental protection has gained considerable attention from numerous professional fields. In hot and humid climates, such as that in Taiwan, people consume a significant amount of energy in the pursuit of a comfortable indoor environment. Considering that the clothing and activity levels of the occupants of a given space cannot be changed, this study aimed to reduce energy consumption while satisfying the demand for comfort.

The indoor comfortable environmental temperature, suggested by American Society of Heating, Refrigerating, and Air Conditioning Engineers (ASHRAE) for summer and winter is environmental temperature (ET) 24.4°C and 21.7°C, respectively [1–3]. The relative humidity (RH) recommended by the ISO 7730 considering thermal comfort parameter is 30–70%. An RH of 50% is regarded as the most comfortable humidity level for a thermal environment. Concerning carbon dioxide, the World Health Organization (WHO) has recommended that indoor carbon dioxide concentrations should be maintained below 1000 ppm [4, 5]. An office building with standard quality air conditioning has a carbon dioxide concentration of approximately 400–700 ppm. The U.S. Environmental Protection Agency (USEPA) recommends that people are not continuously exposed to carbon dioxide concentrations higher than 1000 ppm (1.8 g/m^3^) [6–8]. Under certain temperature and humidity conditions, wind intensifies the dissipation of heat from the human body and reduces the perceived air temperature. Every 1 m/s increase in air velocity reduces the perceived air temperature by 2-3°C [9]. Increased air velocity and thin surface air layers accelerate the dissipation of heat on surfaces, shortening the gap between surface and air temperatures; this is also applicable to skin surfaces; thus, in these conditions, people feel cold and uncomfortable. Thermal comfort in temperatures is related to not only the temperature but also the air velocity. The ISO 7730 standard suggests that, to obtain a perceived moderate air temperature and the most comfortable thermal environment, the average air velocity should be 0-1 m/s and the instantaneous air velocity below 0.15 m/s, with an RH of 50%. Based on these standards, this study analyzed and evaluated indoor environmental temperature, relative humidity, carbon dioxide concentrations, and air velocity to examine the effects of all these parameters on indoor workers [10–12].

Endeavoring to establish a theoretical construct for a healthy and comfortable indoor office environment, we monitored the thermal comfort level and indoor air quality of an air-conditioned office space incorporated with make-up air units (MAUs). In addition to monitoring the physical environment, then, a questionnaire survey of people who worked in an office is conducted to establish the perceived comfortable environment conditions based on the monitoring and questionnaire survey results. We endeavor to not only implement the concept of energy-saving buildings but also to provide people with a healthy and comfortable office environment. Furthermore, from the perspective of an energy manager and by continuously monitoring the system equipment and recording energy consumption data, we can provide a reference of reliable system improvements for energy managers or maintenance workers.

## 2. Experimental Section: The Description of Field Test Office

Field test site for this research is an anonymous office building in Taichung City. The climate of Taichung City, which is in a subtropical zone, is hot and humid. In the summer and autumn months, Taichung experiences abundant rainfall and numerous typhoons; in the winter months, cold weather is introduced by the Northeast monsoon. According to statistics provided by the Central Weather Bureau, in recent years, the average temperature in Taichung has ranged between 12.5 and 29.0°C. Outdoor temperature in Taichung is about a high of 34.3°C and a low of 24.5°C, for a temperature difference of 9.8°C. The highest monthly average temperature occurs in August; the lowest monthly average temperature occurs in January. The number of hours of sunshine per month ranges between 137 and 204, for a monthly average of 170 hours. The annual precipitation is approximately 1800 to 3320 millimeters, with the least amount falling in November and the highest amount falling in June. The annual average relative humidity (RH) ranges between 64 and 77%.

**Figure 1:**
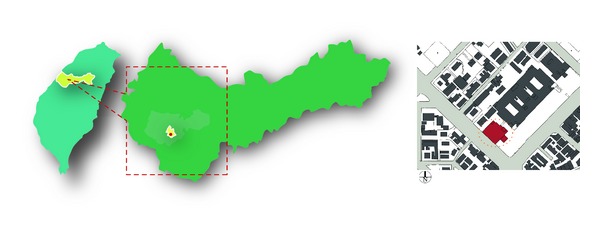
The field test site for this research.

**Figure 2:**
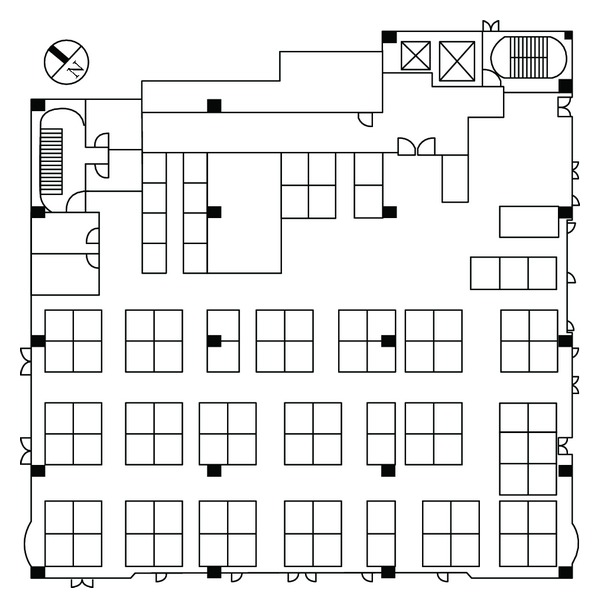
The floor plan of field test for this research.

The research site, shown in Figure 1, is an old building with eight floors, six of which are above ground level, and the remaining two are below ground level. The floor area for this office space is 33.2 m × 29.7 m, that is, 986.04 m^2^, and accommodated 96 office workers. Windows are installed in the west-, south-, and east-facing walls of the building. On the north-facing side of the building, ventilation windows are only installed in the stairwell. The building interior includes various spaces such as offices, conference rooms, reception rooms, and tearooms. We monitor the indoor air quality of an open-plan office space located on the fourth floor with partitions for a tearoom and a photocopy room. The floor plan of this test site is shown in Figure 2. Besides, heating, ventilating, and air-conditioning (HVAC) system of this building is the constant air volume (CAV). In a simple CAV system, the supply air flow rate is constant, but the supply air temperature is adjusted to meet the thermal loads of a space.

## 3. Measured Data of This Original Building 

### 3.1. Temperature, Humidity, and Comfortable Indoor Temperature Conditions

According to statistics provided by our monitored data from summer and winter seasons, the outdoor average temperature in Taichung is 27.6°C, with a high of 34.3°C and a low of 24.5°C, for a temperature difference of 9.8°C in summer. It is significantly higher than the effective temperature for the summer, that is, ET 24.4°C Then, the outdoor average temperature is 12.5°C, with a high of 15.8°C and a low of 10.8°C, for a temperature difference of 5°C for winter. It is significantly lower than the effective temperature for winter, that is, ET 21.7°C, as suggested by ASHRAE. Therefore, indoor thermal comfort must be achieved through the building design. The average relative humidity is about 65.4% and 45.8% in summer and winter, respectively.

### 3.2. Indoor Carbon Dioxide Concentrations and Air Velocity

**Figure 3:**
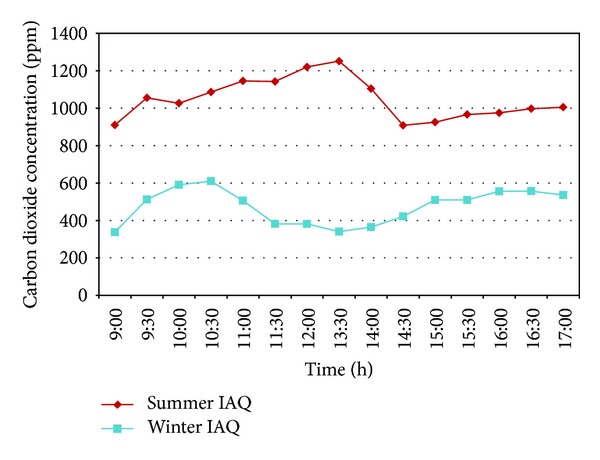
The distribution of measured indoor carbon dioxide concentrations in summer and winter, respectively.

As shown in Figure 3, the measurements, obtained in the summer, generally show a higher carbon dioxide concentration of 910–1200 ppm, which is 210–500 ppm higher than the average concentration of an office building with standard-quality air conditioning. From 09:30–14:00 and at 17:00 on the day of the experiment, the carbon dioxide concentrations exceed 1000 ppm. The highest concentration measured is as high as 1251 ppm, indicating extremely poor air quality. The measurements, obtained in the winter, show carbon dioxide concentrations ranging between 170 and 639 ppm, which correspond to the standard and are significantly lower than the measurements obtained in the summer. As indicateed by the measurements of indoor carbon dioxide, the trend of indoor carbon dioxide concentrations is related to the flow of people in the office. The concentration is higher around 10 a.m. when more people occupied the office for work and business. After 12:00, the concentration gradually declines because few people remain in the office during lunch break. At approximately 14:00, the concentration slowly increases again until 17:00. 

**Figure 4:**
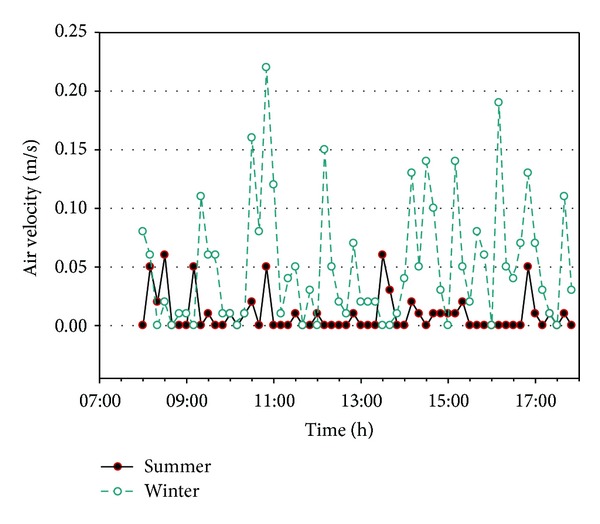
The variation of hourly indoor air velocity in summer and winter, respectively.

Figure 4 shows the measurements of hourly indoor air velocity. The air velocity in the summer ranges between 0 to 0.06 m/s. Furthermore, no significant change in air velocity is measured, and the air flow is difficult to perceive. The air velocity and higher indoor carbon dioxide concentrations in the summer may be the cause of poor ventilation. In the winter, the air velocity was 0–0.22 m/s, with an inconstant instantaneous air velocity that generally remained below 0.1 m/s. The indoor carbon dioxide concentrations in the winter are significantly lower than those in the summer.

The average of air velocity is 0.01 m/s in summer and 0.05 m/s in winter. The average carbon dioxide concentration is 1047.7 ppm in summer and 447.9 in winter. The changes in indoor temperature, average relative humidity, air velocity, and carbon dioxide concentrations are shown in Table 1.

## 4. Questionnaire Surveys

 In addition to evaluating the physical environment, spaces, and mechanical equipment, questionnaire surveys with the occupants of the office are conducted to examine any related issues during the summer and the winter, including the proportion of workers suffering from sick building syndrome caused by carbon dioxide and their assessment of perceived environments.

### 4.1. Questionnaire Survey on Sick Building Syndrome

The questionnaire survey results are used to thoroughly understand the influence that carbon dioxide has on humans. The invalid questionnaires (office workers who experienced allergy, asthma, or cold symptoms, whose average sleep duration is less than 6 hours or who work night shifts) are excluded in this questionnaire survey. A 30.8% of the valid questionnaires exhibit two or more symptoms, related to carbon dioxide concentrations, at a frequency of two days or more per week in the summer. Furthermore, 25.6% of the valid questionnaires (26 to 58 years of age) exhibit two or more symptoms in the summer, related to carbon dioxide concentrations, at a frequency of three days or more per week. A 23.4% of the valid questionnaires exhibit two or more symptoms, related to carbon dioxide concentrations, at a frequency of two days or more per week in the winter. Furthermore, 6.3% of the valid questionnaires (26 to 58 years of age) exhibit two or more symptoms, related to carbon dioxide concentrations, at a frequency of three days or more per week. The office has poor ventilation, which results in the office workers experiencing headaches, stuffiness, dizziness, poor memory, insufficient attention, tinnitus, weariness, drowsiness, and chest tightness. Therefore, the poor ventilation of this office should be improved to build better indoor air quality.

### 4.2. Questionnaire Survey Regarding the Environment

This survey is conducted to understand the environmental satisfaction and provides the suggestions with improvement directions of the occupants of the space. The questionnaire results indicate that most participants are very dissatisfied with the ventilation (33.3%), somewhat dissatisfied with the air quality (28.2%) and the temperature (28.2%), and believe that they can smell mold (10.2%). During the winter, most of the participants are also very dissatisfied with the ventilation (38.3%), dissatisfied with the air quality (29.8%), and believe that they can smell mold (50%).

The participants' satisfaction with the indoor environment perceived is measured through self-evaluation of items such as indoor ventilation, air quality, temperature, and humidity using a 7-point scale. The scale includes the options *very dissatisfied* (−3), *dissatisfied* (−2), *somewhat dissatisfied* (−1), *neither satisfied nor dissatisfied* (0), *somewhat satisfied* (1), *satisfied* (2), and *very satisfied* (3). Indoor thermal comfort is also measured using a 7-point scale, that is, *cold* (−3), *cool* (−2), *slightly cool* (−1), *comfortable* (0), *slightly warm* (1), *warm* (1), and *hot* (3). The mean scores of the collected valid questionnaires are shown in Table 2. The mean scores for the summer indicated greater comfort compared to those for the winter, although most of the office workers felt slightly dissatisfied, listed in Table 2.

The questions regarding indoor thermal comfort results show that, in the summer, 23.1% of the participants selected *slightly cold*, 30.8% selected *comfortable*, 17.8% selected *slightly warm*, 2.6% selected *warm*, and 17.9 selected *hot*, with a total of 38.4% selecting options above *slightly warm*. In the winter, 8.2% of the participants selected *slightly cool*, 12.2% selected *comfortable*, 34.7% selected *slightly warm*, 18.4% selected *warm*, and 20.4% selected *hot*, with a total of 76.5 selecting options above *slightly warm*. In the summer, 30.8% of the participants felt *comfortable*, which was higher than the 12.2% in the winter. Furthermore, in the summer, 23.1% of the participants selected *slightly cool* regarding the indoor thermal comfort, and most of the winter participants (34.7%) selected *slightly warm* regarding the indoor thermal comfort (Table 2). Comparing the office measurements, the air conditioner's operating time is increased in summer because the office workers feel more comfortable in a lower temperature environment. This implies that people living in hot-humid climates are more accepting of the cold and tend to prefer lower environmental temperatures.

To measure the participants' environmental perceptions, an independent samples *t*-test with a confidence interval of 95% is performed regarding the indoor ventilation, air quality, temperature, humidity, and thermal comfort.  Table 3 shows the tests results, where indoor humidity and thermal comfort achieve significant differences (*P* < 0.05). Based on the statistical results, office workers experience indoor thermal comfort in the winter. The humidity level is more satisfactory during the winter than in the summer (Table 3).

The analysis results of the indoor conditions reveal that, in the summer, 30.9% of the participants selected ventilation, 29% selected temperature, and 27.2% selected air quality; in the winter, 43.3% of the participants selected ventilation, 31.3% selected air quality, and 11.9% selected temperature. Summarizing the results, most of the office workers were dissatisfied with the indoor ventilation and hoped for improvements in the future. Although the indoor air velocity in the winter was higher than in the summer, the air quality satisfied the standard and could be improved by adjusting the temperature and return air rate, or increasing the import of fresh air.

## 5. Improved Strategy for This Old Building 

**Figure 5:**
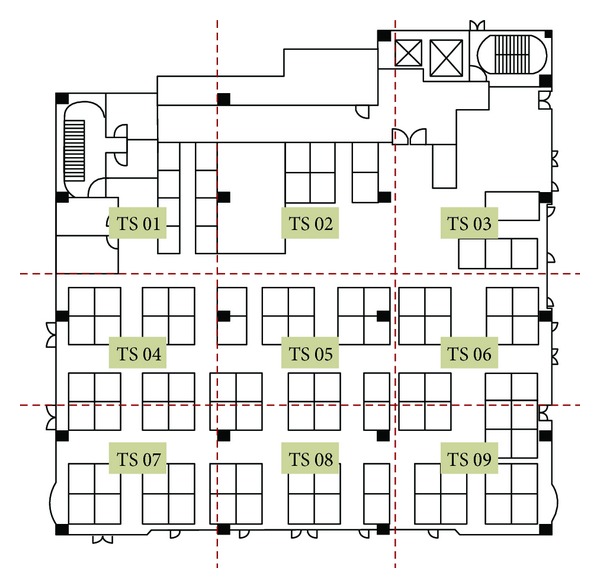
Nine sections for monitoring the distribution of CO_2_ concentrations.

To raise the indoor comfortable environment, the distribution of carbon dioxide concentrations in the office is examined. This measured office is divided into nine sections, each of which measured 9 m × 11 m, 99 m^2^, shown in Figure 5. 

**Figure 6:**
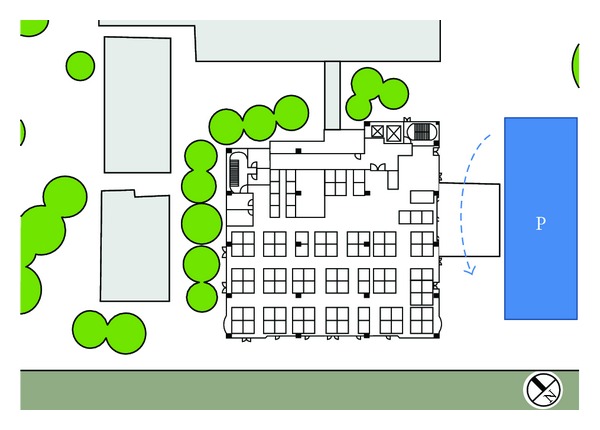
The configuration of outdoor environment.

In order to maintain the air temperature at comfortable temperature, the variable air volume (VAV) system with the characteristics of supply air volume flow rate to match the variation of the pace cooling is adopted to improve the indoor air quality. Then, the energy-saving potential of this VAV system is investigated in this study. Otherwise, the characteristics of outdoor environment should be considered to select suitable positions to install air intakes to improve the indoor air quality. The building entrance is located on the southeast side near to a parking lot, adjacent to a driveway; thus, the imported air may be polluted by traffic. Otherwise, banyans had been planted near section of TS 01, 04, and 07 to separate road lanes. The configuration of outdoor environment is shown in Figure 6. 

Then, in order to monitor the energy-saving effects of this proposed strategy, we categorized air-conditioning energy conservation and improvement in the following three parts: (1) the installation of inverters and cold-water air conditioners; (2) the installation of three PAHs with 24 outputs, and the allocation of 5 periods for increasing the indoor air exchange rates and reducing discomfort; and (3) the installation of a building energy management system (BEMS) to monitor the energy consumption of cold-water air conditioners, cooling towers, and compressors to control the energy efficiency of systems and enhance energy saving rates.

## 6. Experimental Results and Discussions

### 6.1. Indoor Temperature and Humidity

The measured indoor temperature ranges between approximately 21.2 and 23.8°C, which is 0.6–3.2°C lower than ET 24.4°C, suggested by ASHRAE. Because the air-conditioner designer has considered the high number of occupants in the space and the frequent flow of people, the air-conditioner is used more frequently and sets to a lower temperature to respond to a higher number of occupants during working hours. Consequently, slower flow of people or fewer office occupants result in a relatively lower indoor temperature. In the winter, the measured temperature ranges between 19.7 and 21.5°C, near ET 21.7°C, suggested by ASHRAE. Therefore, our test space has a comfortable temperature. 

**Figure 7:**
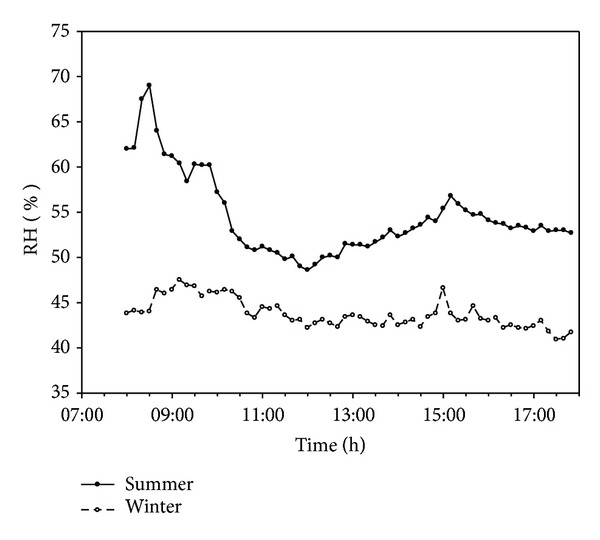
The hourly indoor humidity difference between summer and winter, respectively.

The indoor relative humidity (RH) measured results of this study show indoor RH ranging between 48 and 62% in the summer, where higher humidity is measured before 10 a.m., and a humidity of between 50 and 55% is measured after 10 a.m. In the winter, the indoor humidity ranges between 41 and 46%, and no significant changes in humidity are observed, shown in Figure 7.

### 6.2. Improvement of Indoor Air Quality

**Figure 8:**
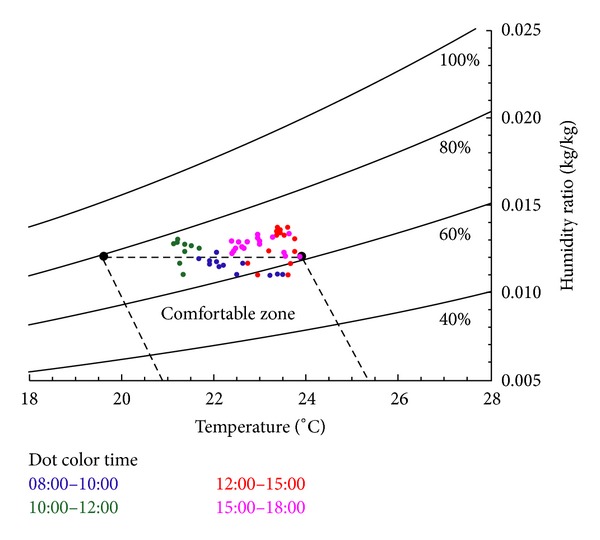
Dot distribution of the environment temperature, humidity, and thermal comfort in the summer before improvement.

The measured distributions of carbon dioxide concentrations in the office are obtained during the time intervals where the carbon dioxide concentrations are the highest. A scientific illustration software program Surfer Suite 8.0 3D is used to plot carbon dioxide concentration distribution, shown in Figure 8. Sections TS 08, 02, and 03 of the office have higher concentrations because a significant number of people used the doorway, and the three photocopiers are also positioned in these sections. The frequent flow of people and electronic equipment operations increased the concentrations in these two sections. By contrast, TS 04 and 07 are the sections with the lowest concentrations. The flow of people is minimal near the northwest of the building, which is the back of the building. Therefore, to improve the indoor air quality, the outdoor air on TS 04 and 07 can be led into indoor via air interchanger.

### 6.3. Comprehensive Analysis of Indoor Thermal Comfort

**Figure 9:**
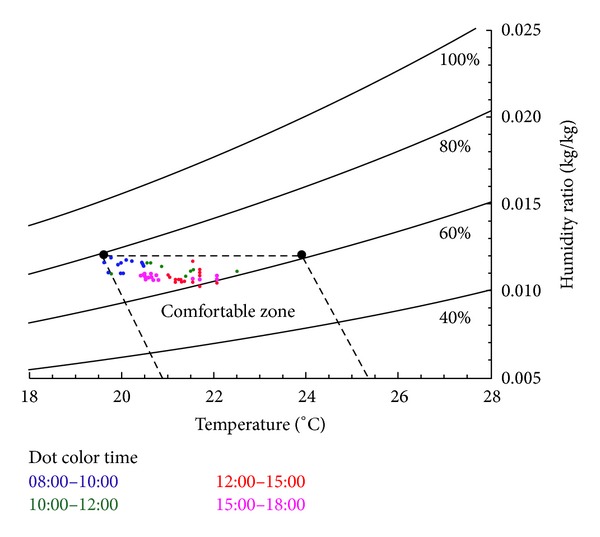
Dot distribution of the environment temperature, humidity, and thermal comfort in the summer after improvement.

Using the comfort range specified in the ASHRAE standard 55–2004 (Thermal Environmental Conditions for Human Occupancy) to analyze the measured indoor temperatures, humidity, and thermal comfort, the results indicated that 32% of the period monitored in the summer corresponded to the standard comfort zone, whereas the remaining uncomfortable conditions were induced by excessive humidity before improvement, shown in Figure 8. After the installed improved air-conditioner devices, 95% office workers corresponded to the standard comfort zone, shown in Figure 9.

### 6.4. Energy Management and Strategies

**Figure 10:**
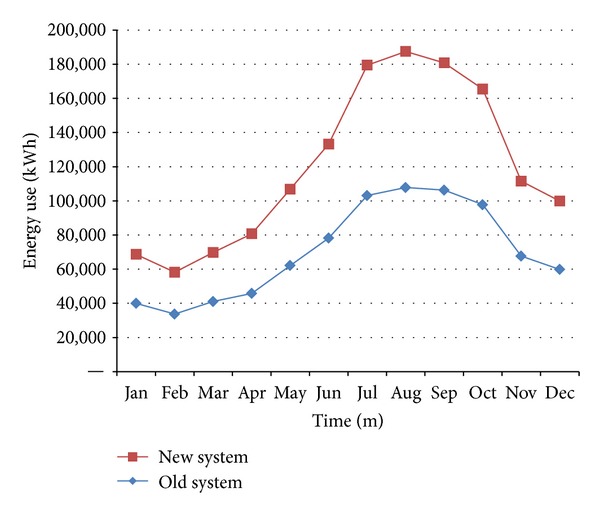
The electricity consumption of existing and improved air-conditioning systems.

**Figure 11:**
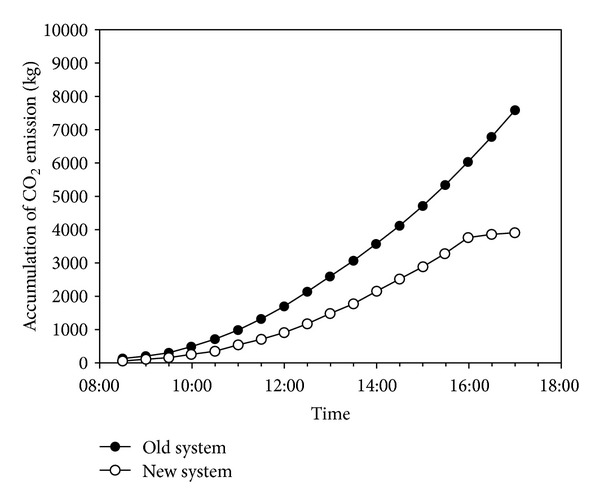
CO_2_ emission reductions per day.

The air-conditioning energy conservation and improvement have been categorized in the following three parts: (1) the installation of inverters and cold-water air conditioners; (2) the installation of three PAHs with 24 outputs and the allocation of 5 periods for increasing the indoor air exchange rates and reducing discomfort; and (3) the installation of a building energy management system (BEMS) to monitor the energy consumption of cold-water air conditioners, cooling towers, and compressors to control the energy efficiency of systems and enhance energy saving rates. According to the energy saving rates measured by electricity monitoring, the suggested improvements could save 244936 kWh every year (Table 4 and Figures 10 and 11).

The energy conservation and improvement project proposed in this study entails the improvement of the air-conditioning system in the research building, including the cold-water chiller, cooling tower, water circulating pump improvements, and control valve upgrades.

## 7. Conclusion

This study considered both the perceptions and physical aspects of the environment by measuring the physical environment parameters and conducting questionnaire surveys. There are some conclusions summarized as follows.

1. Although the indoor temperatures measured in the summer were significantly lower than the comfortable temperature range, the occupants expressed dissatisfaction in a survey of their environmental perceptions. Additional factors, such as the ventilation or crowdedness, may have influenced the occupants' perception of indoor thermal comfort. Otherwise, the occupants reported discomfort because of the poor air quality (odors), limited space, and excessive electronic equipment.
2. Because Taiwan's climate is humid and hot, a temperature within the comfortable zone still caused the participants discomfort. The intensity of the indoor air velocity also influenced their perceptions of comfort.
3. The monitoring result of indoor air velocity in this study reveals that it is not higher than the requirement, denoting it needing better ventilation. The survey results show that the participants are very dissatisfied with the indoor air quality, expressing the need to improve ventilation. This study shows that most people believe that more fresh air increases the degree of comfort, and people still demand a higher air exchange rate or higher air quality even when the indoor air quality is above moderate.
4. According to the analysis results of comfort range based on ASHRAE standard 55–2004, the office workers correspond to the standard comfort zone increases from 32% to 95% after improving this old building.
5. By constructing and analyzing an energy management system, the analysis results reveal that the installation of PAHs can increase air exchange rates and the degree of comfort. The installation of cold-water air conditioners with inverters can enhance efficiency and save energy. Furthermore, monitoring the electricity consumed by the cold-water air conditioners, cooling tower, and compressors using a BEMS can control the system's energy efficiency and enhance energy-saving rates.

## Figures and Tables

**Table 1:** Measured results for the indoor and outdoor physical parameters of the office.

Time of measurement	Outdoor		Indoor			
	Average temperature	Average RH	Average temperature	Average RH	Air velocity	CO_2_ concentration
	(°C)	(%)	(°C)	(%)	(m/s)	(ppm)
Summer	27.6	79.8	25.8	65.4	0.01	1047.7
Winter	12.5	70.4	20.6	45.8	0.05	447.9

**Table 2:** Mean scores for environmental satisfaction.

	Indoor ventilation	Indoor air quality	Indoor temperature	Indoor humidity	Indoor thermal comfort
Summer	−1.3	−1.2	−0.8	−0.2	0.4
Winter	−1.7	−1.7	−1.1	−0.7	1.1

**Table 3:** Results of the independent samples *t*-test for the summer and the winter.

	Summer	Winter	*t* value	*P* value
Ventilation	2.72 ± 1.36	2.27 ± 1.36	1.522	0.132
Air quality	2.79 ± 1.38	2.24 ± 1.21	1.949	0.055
Temperature	3.23 ± 1.37	2.82 ± 1.42	1.339	0.184
Humidity	3.79 ± 1.08	3.29 ± 1.20	2.019	0.047
Thermal comfort	4.36 ± 1.58	5.13 ± 1.38	−2.401	0.019

**Table 4:** Cost of electricity for office buildings.

	Time of operation (h)	Average electricity consumption (kW)	Cost of electricity (NT/8.5 hr)	Accumulated CO_2_ emissions (kg)	CO_2_ reduction (kg)
Existing systems	8.5	164	5880	7,580	Nil
Newly installed systems	8.5	88	3150	3,893	3,687

(1) CO_2_ emissions per unit of electricity consumed = 0.638 kg-CO_2_/kWh (TPC, 2007).

(2) Cost of electricity: kWh = 0.14 (NT$).
